# Peeking into the Femtosecond Hot-Carrier Dynamics Reveals Unexpected Mechanisms in Plasmonic Photocatalysis

**DOI:** 10.1021/jacs.3c12470

**Published:** 2024-01-10

**Authors:** Giulia Dall’Osto, Margherita Marsili, Mirko Vanzan, Daniele Toffoli, Mauro Stener, Stefano Corni, Emanuele Coccia

**Affiliations:** † Dipartimento di Scienze Chimiche, Università di Padova, via F. Marzolo 1, 35131 Padova, Italy; ‡ Dipartimento di Fisica e Astronomia “Augusto Righi”, University of Bologna, Viale Berti Pichat 6/2, 40127 Bologna, Italy; § Dipartimento di Fisica, University of Milan, Via Giovanni Celoria 16, 20133 Milano, Italy; ∥ Dipartimento di Scienze Chimiche e Farmaceutiche, University of Trieste, via L. Giorgieri 1, 34127 Trieste, Italy; ⊥ Istituto Nanoscienze-CNR, via Campi 213/A, 41125 Modena, Italy

## Abstract

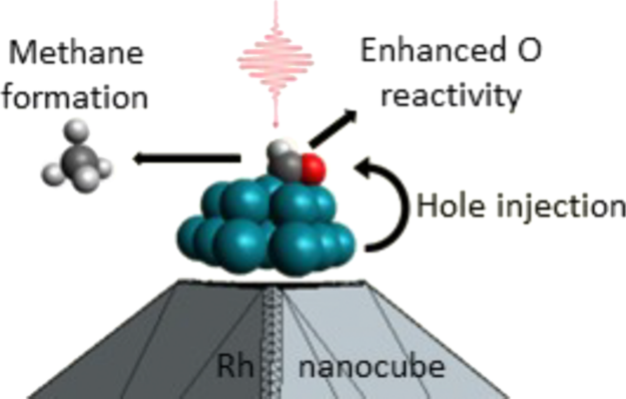

Plasmonic-driven photocatalysis may lead to reaction selectivity that cannot be otherwise achieved. A fundamental role is played by hot carriers, i.e., electrons and holes generated upon plasmonic decay within the metal nanostructure interacting with molecular species. Understanding the elusive microscopic mechanism behind such selectivity is a key step in the rational design of hot-carrier reactions. To accomplish that, we present state-of-the-art multiscale simulations, going beyond density functional theory, of hot-carrier injections for the rate-determining step of a photocatalytic reaction. We focus on carbon dioxide reduction, for which it was experimentally shown that the presence of a rhodium nanocube under illumination leads to the selective production of methane against carbon monoxide. We show that selectivity is due to a (predominantly) direct hole injection from rhodium to the reaction intermediate CHO. Unexpectedly, such an injection does not promote the selective reaction path by favoring proper bond breaking but rather by promoting bonding of the proper molecular fragment to the surface.

## Introduction

Light is well-known to be an eclectic reagent, catalyst, and possible product in chemical reactions.^1−3^ Its interaction with nanostructures has further expanded the possibility of using light to manipulate chemical systems with extremely high precision and accuracy and, in turn, could affect many relevant technological fields such as sensing, catalysis, renewable energy, communication, and medicine.^4−13^ Among all possible processes appearing at these scales, the activation of the Localized Surface Plasmon Resonances (LSPR) is one of the most peculiar and in the past decades its theoretical comprehension already gave notable outcomes.^9,14−19^ A particularly interesting and technologically relevant feature arising from the activation of the LSPR resides in the use of the energy released by its decay with a host of potential applications.^20−22^ LSPR decay can be summarized through the following stages: following its excitation, the collective oscillation of the electronic cloud starts to dephase (Landau damping), resulting in the formation of electron–hole pairs, neutral excitations that store the energy originally absorbed by the plasmon. Such nonequilibrium state of excited electrons and holes rapidly thermalizes, resulting in a configuration where the carriers (electrons and holes) follow a Fermi–Dirac distribution at a higher temperature with respect to the lattice one, as if the electronic system was heated up. The electronic system remains “hot” until the electron–phonon scattering transfers all the extra electron energy to the lattice, getting further dissipated via thermalization to room temperature.^23−32^ Mechanisms for hot-carrier production were studied both experimentally and theoretically over the past years,^33−41^ but, despite the attention such a problem attracted recently,^40,42−56^ the ways hot carriers mediate chemical reactions driving the photochemistry at the surface of metallic nanoparticles (NPs) are still largely unknown. Indeed, once generated, hot carriers may interact with other species in proximity of the metallic NP, like a solid semiconductor or a molecule, in the latter case directly triggering chemical reactions. Several groups, in fact, were able to harness plasmon-generated hot carriers to perform different reactions, usually with higher selectivities and rates as compared with their thermal counterparts. Some of these catalyzed reactions, such as nitrogen fixation and water splitting have a remarkable application potential, also considering the global environmental and societal issues posed by global warming and climate change, so that plasmon-driven photocatalysis could play a role toward a green and sustainable future.^57−67^ In this framework, considering the dramatic impact that carbon dioxide has on our environment, a lot of work is nowadays devoted to control reactions that allow efficient CO_2_ conversion to methane or other short chain hydrocarbons, important in a circular economy perspective.^68−73^ In 2017, Zhang et al. demonstrated that in the presence of rhodium nanocubes (with a side length of 37 nm) and a hydrogen-rich environment, CO_2_ is reduced to a 60:40 ratio mixture of CH_4_ and CO. However, in the presence of an external electromagnetic pulse matching the optical absorption of the nanocubes (a LSPR band in the UV broadened by extensive interband excitation^74,75^), the selectivity toward CH_4_ rises to values above 90%.^76^ The effect was tentatively assigned to hot-electron injection in antibonding orbitals of a reaction intermediate (adsorbed CHO^76−79^) based on ground state density functional theory (DFT) calculations for CHO adsorbed on a model Rh(100) surface. Even though many mechanisms have been proposed for CO_2_ reduction in general, there is a consensus in literature to consider CHO as reaction intermediate for CO and CH_4_ generation on a rhodium surface.^80,81^ However, the complexity of the system calls for a more refined theoretical approach. Indeed, to adequately study such a complex photocatalytic phenomenon it is necessary to consider the presence of all the actors, namely, the molecule, the NP and the external electromagnetic pulse.^82−84^ All of those elements have to properly interact to account for the time-dependent dynamics of the hot carriers. Moreover, the electronic structure of the molecular moiety needs to be described accurately enough to consider the intrinsic quantum (QM) nature of the physical system. Indeed, even considering the sole interaction between hot carriers and molecular species, plasmon enhancement of reaction selectivity might be caused by different processes such as charge transfer, lowering of reaction barriers in excited state pathways, near fields enhancement of intramolecular transitions, activation of specific molecular vibrations, or simply thermal effects as the local temperature in proximity of the NP might exceed the ambient one.^35,85−91^ The size of the system (tens of nm) and the span of involved time scales (from fs to hundreds of ps) hamper a full-QM treatment of the entire system, and thus, other computational approaches are needed.^25,36^

Recently, our group has developed a multiscale methodology to deal with electronic dynamics of molecules close to metal NPs,^92−95^ encompassing post-DFT approaches such as GW + Bethe–Salpeter equation (BSE)^96^ whose accuracy for electronic excitations at interfaces is well documented.^97^ Here, we apply such a methodology to clarify the origin of the photoenhanced selectivity of CO_2_ reduction by H_2_. In particular, we perform extensive simulations of the electronic dynamics of the system, looking for possible charge transfer effects. Moreover, we analyze the effects these charge transfers have on the electronic density of the systems and the related consequence on the reactivity.

The results show that it is hole injection that speeds up the pathway leading to CH_4_ production, by making the CHO fragment’s oxygen more reactive, an apparently counterintuitive results for a reduction reaction. We could also explore the microscopic mechanism of the injection, showing that direct photoinduced charge transfer is prevailing for the nanocube thanks to the electromagnetic enhancement effect associated with the metal NP electronic resonance.

## Results and Discussion

**Figure 1:**
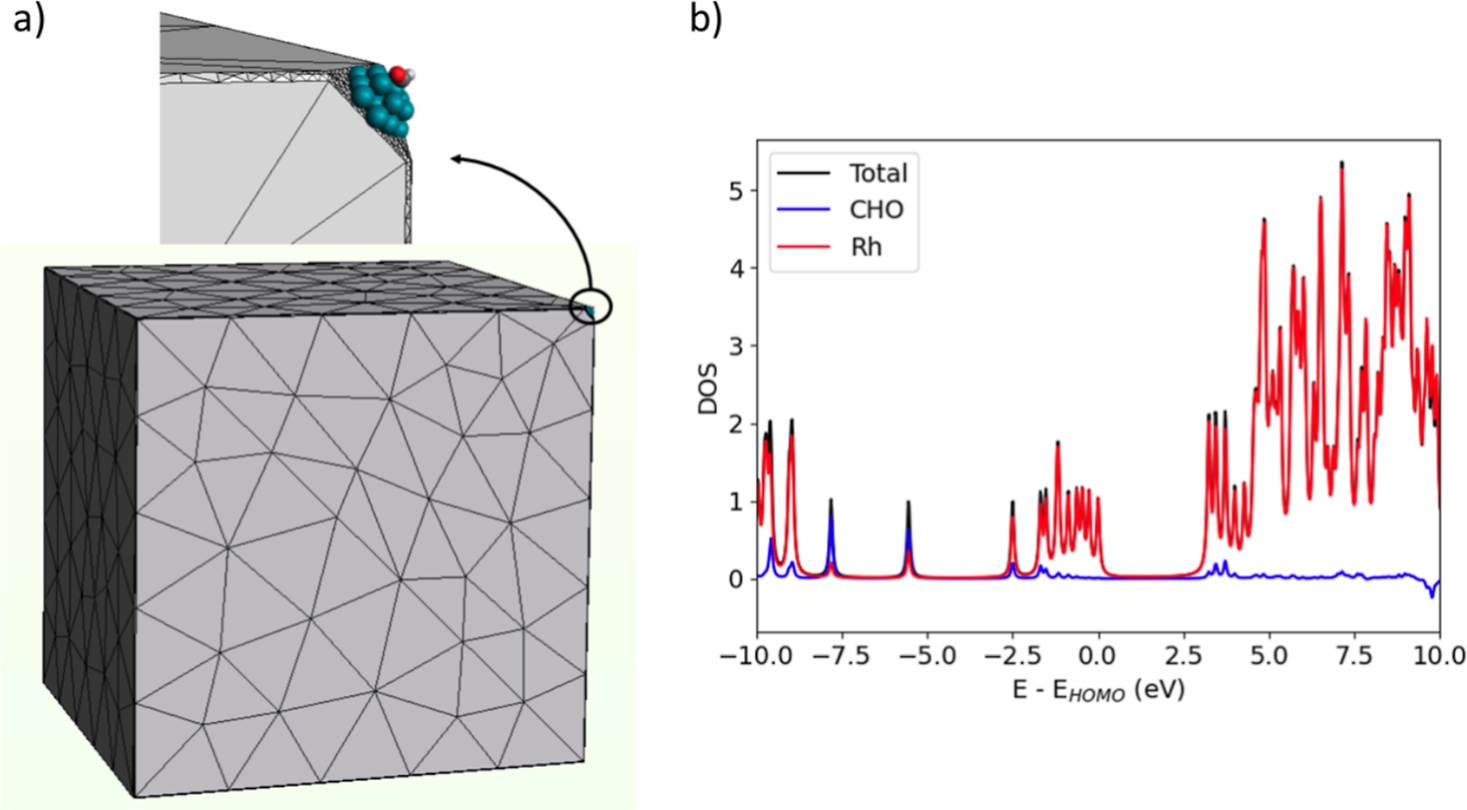
(a) Multiscale model of the system composed of Rh nanocube, Rh cluster and CHO; (b) ground-state GW-calculated density of states (DOS) of QM portion before the pulse is switched on. *E*_HOMO_ is the energy of the highest occupied molecular orbital (HOMO).

A multiscale approach has been applied to study the electron dynamics driven by an external pulse and assisted by plasmonic effects into the CHO adsorbed on a Rh nanocube, which is the experimentally relevant system.^76^ In our simulations, the Rh NP is represented as a continuous dielectric nanocube of 37 nm edge, as in the experiment, characterized by the empirical Rh dielectric function ϵ(ω),^94^ and edge curvature equal to Rh atomic radius. One of the vertices of the nanocube is cut perpendicular to the (111) direction. Above the cut, at the atomic interlayer distance, an atomistic Rh_19_ cluster is set, mimicking the vertex itself as shown in panel (a) of Figure 1. Adsorbed to the cluster is a CHO fragment, being the CHO dissociation into CH + O the rate-determining step in the thermally activated reaction toward CH_4_.^98,99^ The atomistic portion of the composite system has been described at the QM level, while a classical polarizable continuum model (PCM) has been used for the large remaining part of the NP. QM calculations are based on DFT, GW, and BSE. All the results of this work have been obtained considering clamped nuclei and therefore focusing on the electronic degrees of freedom, on a time scale of tens of femtoseconds. Larger timeframes would require the nuclear dynamics to be explicitly included in the simulations. We decided for this multiscale partition of the whole system because it is experimentally known that hot carriers are more effectively generated at the corners of nanostructures,^21,76,100,101^ and that plasmon-mediated reactions take place more efficiently at these hot spots. Details of the theoretical framework are given in Methods, while the strategy used to define the multiscale modeling is provided in the Supporting Information (see Figures S1 and S2 and Table S1). The goal of this work is to rationalize the reaction selectivity toward CH_4_ in the case of a photocatalytic process, as observed experimentally.^76^ In order to accomplish that, we focused on the understanding of three main aspects: (i) the mechanism and nature of charge injection into CHO moiety, the reaction intermediate of the rate-determining step:^99^ dissociation of C–H or C–O bond in CHO leads to carbon monoxide or methane, respectively; (ii) the fate of oxygen and hydrogen atoms along the plasmon-assisted photoinduced hot-carrier dynamics; and (iii) the role of the environment in terms of pure electronic dephasing in the time propagation. In order to accomplish that, we analyzed the results in terms of field-free polarization of the system, of the hot-carrier dynamics in the QM portion alone, and in the whole multiscale system.

### Electronic Structure of the Ground State

The molecular orbitals (MOs) of the adsorbed CHO fragment are strongly mixed with those of the Rh atoms. The GW projected density of states (PDOS) of the CHO + Rh cluster, displayed in panel (b) of Figure 1, shows CO bonding MOs at low energies (around −8 and −6 eV) and, interestingly, populated CO antibonding MOs below the Fermi level between −2.5 and −1.5 eV. Indeed, the C–O bond length for the adsorbed CHO species results to be around 1.27 Å, a larger value compared to the same species in vacuum where the C–O bond is 1.18 Å long, hinting to the capability of Rh to catalyze the C=O bond breaking already in the electronic ground state, i.e., thermally. This is in line with experimental results, where the thermal mechanism does provide reduction to CH_4_, although with limited selectivity. Moreover, photoinduced mechanisms for bond weakening and dissociation in plasmonic systems have been also identified, as, e.g., experimentally observed for gold–thiol bond on the surface of gold NPs.^102^

### Hot-Carrier Dynamics in the QM Portion Alone

Preliminarily, the time-dependent Schrödinger equation (TDSE) has been propagated for the QM portion (Rh_19_–CHO) considered alone (i.e., not interacting with the NP and in absence of any external environment). The dynamics is triggered by a Gaussian-enveloped electric field pulse with a full width at half-maximum (fwhm) of 21 fs, centered at 3.4 eV (365 nm), maximum intensity of 3 × 10^4^ W/cm^2^, and an electric field vector pointing perpendicular to the rhodium cluster layers. The value of 3.4 eV (365 nm), taken from the experimental work, is close to the plasmonic resonance of the rhodium nanocube.^76^ A maximum intensity of 3 × 10^4^ W/cm^2^ was chosen to simulate the hot carrier linear response in the weak-field regime. In the experimental work,^76^ the authors show that the CH_4_ photoproduction rate moves from linear to superlinear when the pulse intensity is increased. It has been suggested that such behavior is an evidence of the role of hot carriers in the photocatalytic process,^58,103,104^ which involves the excitation of vibrational normal modes of the intermediate in the rate-determining step.^91,105^A larger pulse intensity could thus possibly trigger different nuclear dynamics via a multiple-excitation regime.^106^ The study of the reaction QM efficiency as a function of the light intensity is beyond the scope of the present work, which is thus limited to the linear response conditions of hot carrier degrees of freedom. Indeed, we do not compare computed results at different pulse intensity values since we focus on the understanding of the enhanced selectivity rather than on the production rates.

The continuous wave LED light used in the experiment^76^ has a finite coherence time, that sets the limit for the time duration of a coherent QM dynamics like those we are simulating here. In other words, from the point of view of the system excitation, such light can be best approximated as a train of light pulses of relatively low intensity, whose effects sum up incoherently. We simulate the consequence of one such pulses. Based on the known coherence length^107,108^ of such light sources (a few μm, according to eq 1 in ref (109)) and speed of light, a coherence time of tens of fs can be estimated, in line with the fwhm of the pulse adopted in our simulations (21 fs). This result justifies the use of a femtosecond pulse in the calculations.

**Figure 2:**
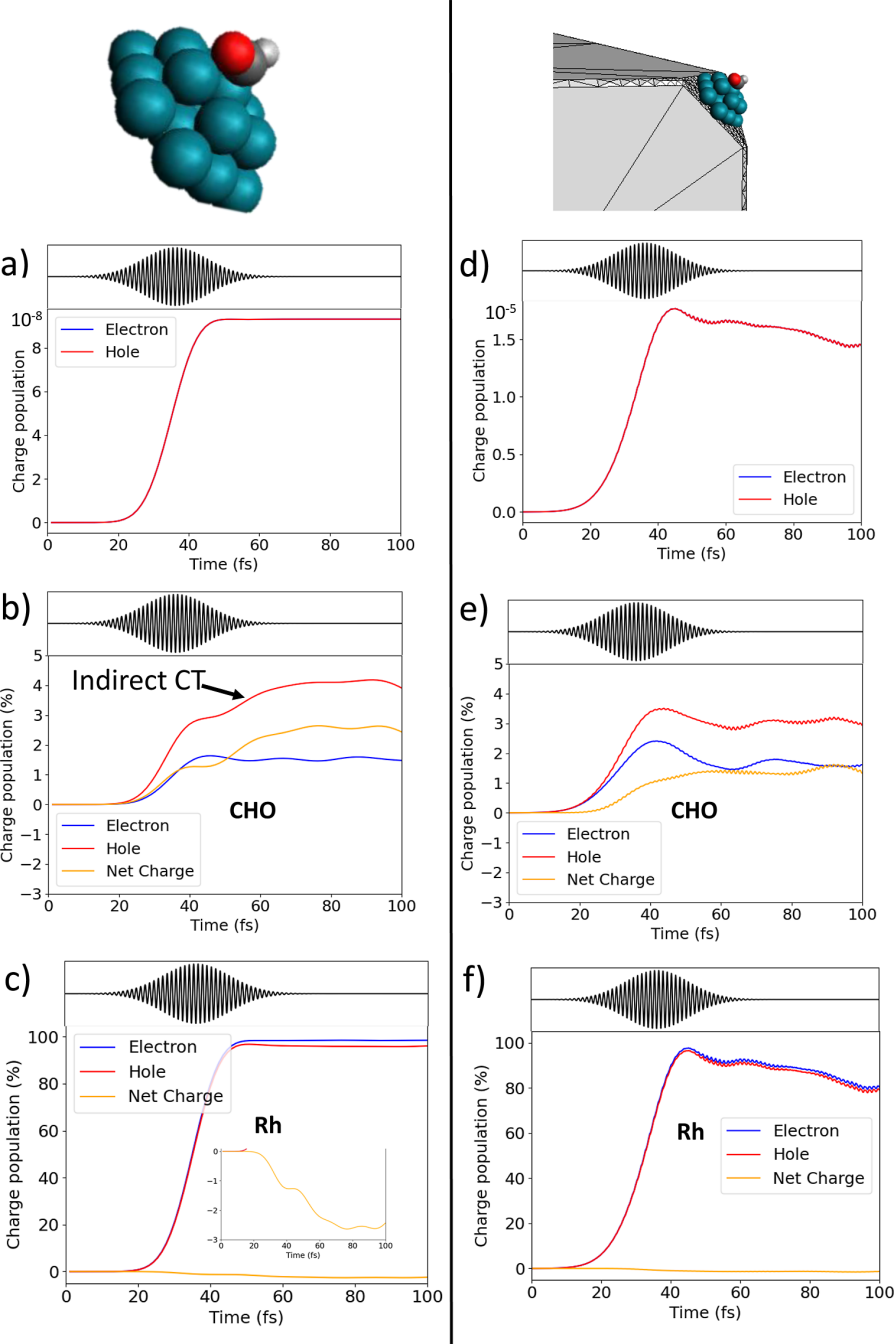
a) Time-evolution of the photoinduced charge carrier populations (electron and hole) into the QM portion of the system (see Figure 1), without the classical portion of the Rh nanocube. (b) Time-evolution of the photoinduced charge carrier population into CHO (%), without the classical portion of the Rh nanocube. (c) Time-evolution of the photoinduced charge carrier population into the Rh cluster (%), without the classical portion of the Rh nanocube. (d) Time-evolution of the photoinduced charge carrier population into the QM portion of the system in the presence of the classical portion of the Rh nanocube; note that scales in panels (a,d) are different. (e) Time-evolution of the photoinduced charge carrier population into CHO (%), in the presence of the classical portion of Rh nanocube. (f) Time-evolution of the photoinduced charge carrier population into Rh (%), in the presence of the classical portion of the Rh nanocube; the photoinduced net charge as a function of time is magnified. Time evolution of the pulse is also shown for the sake of clarity in all panels. The net charge is defined as the difference between the hole and electron percentages.

Panel (a) of Figure 2 reports the time evolution of electron and hole generation in the QM portion when the pulse is switched on. The curves related to electron and hole are plotted with the same sign to show that they are exactly superimposed, as expected, since no other charge source/sink is modeled. The net charge is defined as the difference between hole and electron percentages: a positive (negative) value indicates a defect (accumulation) of electrons. Plotting the percentage of photoinduced charge carrier population in CHO and Rh separately, shown, respectively, in panels (b,c) of Figure 2, one observes a net hole injection into CHO or conversely, a net electron excess into the Rh cluster (see the orange line representative of the net photoinduced charge). Looking at the Rh and CHO hole populations within the first 40 fs of dynamics, the rising of the hole population within the pulse duration shows that a direct charge injection mechanism is in action.

This is in line with general suggestions about the direct injection.^109^ However, this is not the whole story. Between 50 and 60 fs, i.e., after the pulse ended, an increase in the slope of the CHO hole population occurs, concurrently to a Rh hole population decrease (see the zoom of panel c of Figure 2). In other words, there is a net transfer of holes from Rh to CHO after the pulse. This is a signature of an indirect charge generation in which holes, originally excited in the Rh cluster, are transferred to the CHO fragment. To quantitatively estimate the % contribution of indirect and direct charge-transfer mechanisms for the QM cluster alone, we fitted the hole population of CHO and the hole population of Rh with two sigmoid functions until 66 fs. The direct charge transfer contributes 75%, while the contribution of the indirect one is 25% (Figure S3 of the Supporting Information). The indirect mechanism is identified as a concomitant decrease of holes in Rh, and their increase in the fragment. The distinction is based on time scales, which is indeed the discriminant between direct and indirect processes. Details of the fitting procedure are given in the Supporting Information.

The present results confirm that in a realistic system both mechanisms can coexist (but the role of the rest of the NP is still to be discussed in this respect, vide infra). Notably, intramolecular excitation is also at work, generating an amount of electron–hole pairs comparable to that of the net charge injection.

The absolute value of charge injection depends on the external field intensity (linearly in the present regime). Normalizing charge injection to the maximum total charge generation, as done in panels (a,c) of Figure 2, we see that 3% of the generated holes is located on the CHO fragment for the isolated QM portion. The same percentage of electron injection is then found on Rh residue, evidenced by the magnification of the net charge in panel c of Figure 2. This result is in line with previous calculations of incident photon conversion efficiency (IPCE), which achieve values between 2 and 5% at the plasmon resonance peak.^110,111^ IPCE exclusively refers to the efficiency to generate hot carriers with a given pulse intensity.

### Hot-Carrier Dynamics in the Full Multiscale System

After applying an equilibration procedure (see ref (97)), in which the ground state electronic density of the QM portion, i.e., CHO + Rh cluster, is relaxed in the presence of the polarizable continuum describing the classical portion of the system, the coupled equations of motion of the QM and classical portions have been propagated in the presence of the same pulse as before, consistent with the experiments.

Panel (d) of Figure 2 reports photoinduced electron and hole populations as a function of time for the full (QM + classical) system. As in the other panels, the hole and electron injection are represented with the same sign to highlight that the total charge is conserved. Charge generation is around 3 orders of magnitude larger than in the absence of the NP [compare panels (a,d)], an evidence of how the Rh nanocube enhances the effect present in the Rh_19_–CHO cluster. At the same time, the presence of the Rh nanocube modifies the charge generation time profile, providing a quicker charge separation. Clearly, the size of the NP has an effect on both the charge generation and dynamics, as is evident when results of Figure 2 with and without the classical portion of the Rh nanocube are compared.

The electron and hole injection computed for the separate CHO and Rh fragments [panels (e,f)] of Figure 2) shows that only a direct charge transfer is observed in the presence of the classical portion of the Rh nanocube.^112^ Indeed, the presence of the classical NP strongly enhances the direct charge transfer mechanism as a consequence of the local field enhancement, making other charge transfer mechanisms negligible. However, if only a near-field effect of NP were present, one would expect a magnification of the time profile without changes in its shape. Therefore, the NP-driven mechanism is multifactorial, involving near field, change in energy levels, and their accessibility. In fact, the suppression of the indirect charge transfer can be explained by considering that Rh localized holes are electrostatically less stable in the QM system alone than in the full system. The full NP dynamically polarizes as a response to the induced charge carriers, providing a reaction field that makes them more stable and less prone to be transferred to the CHO fragment.

The percentage of charge injection (computed normalizing charge injection to the maximum total charge generation) slightly decreases with respect to the results in the absence of the classical NP, moving from ∼3 to ∼2% in the presence of the additional portion of the NP, but of course, the absolute number of injected holes at the same incident intensity is much higher in the present case (the factor 10^3^ mentioned above). The hot-carrier generation rate of approximately 10^10^ s^–1^ is in line with previous estimates from 10^9^ to 10^17^ s^–1^, according to the nature and size of the NP, and to the intensity of the pulse.^26,101,113−118^

### Investigating the Photoinduced Change in the System Reactivity

**Figure 3:**
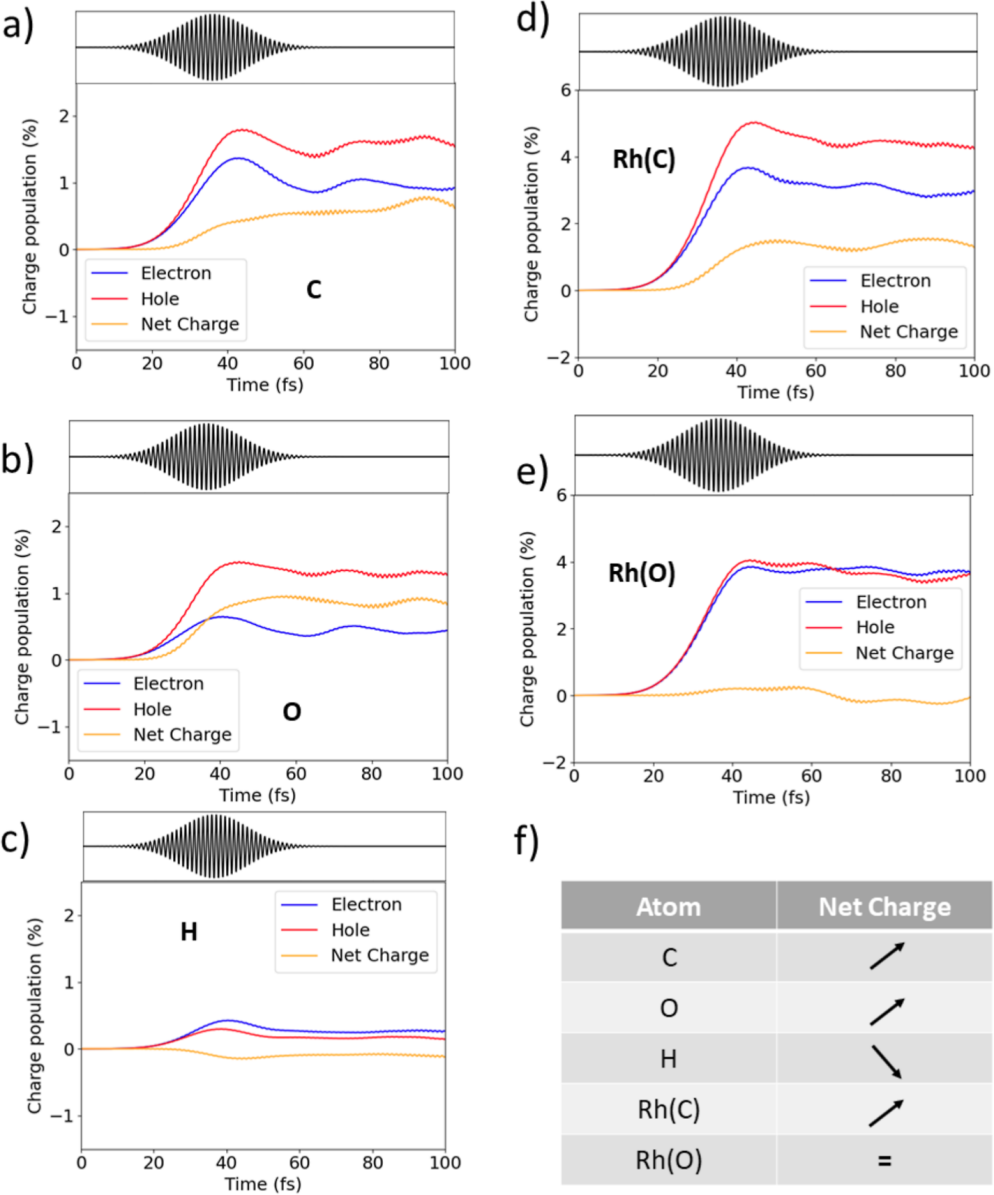
Electron, hole, and net-charge photoinduced population for (a) C, (b) O, (c) H, (d) Rh closest to C, and (e) Rh closest to O, from the coherent electron dynamics performed with the classical portion of the Rh nanocube. Panel (f) summarizes the change of electron charge. The net charge is defined as the difference between hole and electron percentages.

Having assessed the nature of the electronic excitation and its sub-100 fs dynamics, we move now to analyze quantities that may reveal why such electronic dynamics leads to the experimentally determined photoinduced selectivity of the CO_2_ reduction reaction. In panels (a–e) of Figure 3 the dynamics of the charge generated specifically on the C, H, and O atoms is shown, together with the results for the Rh atoms closest to C and O, labeled as Rh(C) and Rh(O), respectively. These results concern the full composite system, i.e., the coupled dynamics of the Rh_19_–CHO cluster and classical nanocube. C and O atoms are affected by a net hole injection [panels (a,b)], while the H atom is characterized by a slight electron injection [panel (c)]. Rh(C) also becomes hole rich, while no appreciable charge change located on Rh(O) is found. All of these features are sketchily summarized in panel (f) of Figure 3.

**Figure 4:**
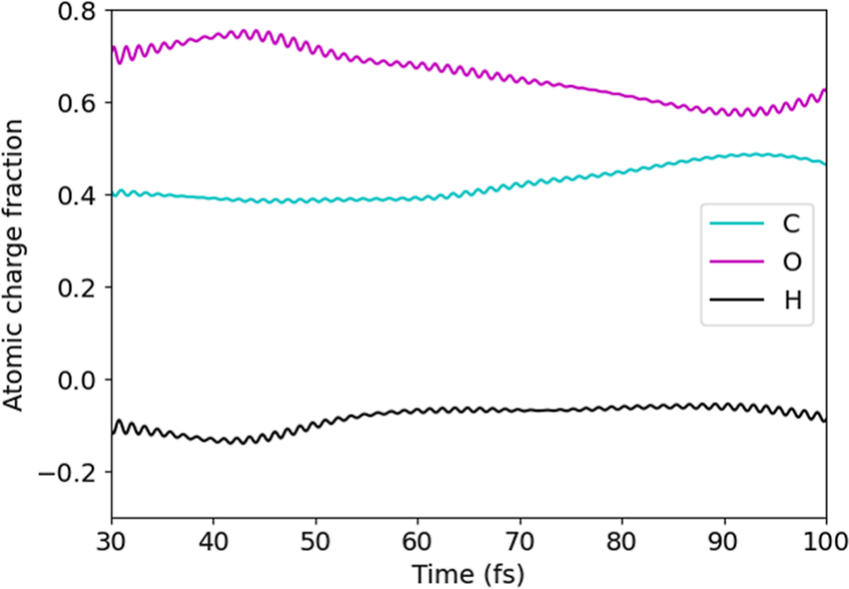
Time-resolved Fukui-like charge for C, O, and H atoms from the coherent electron dynamics performed in the presence of the classical portion of the Rh nanocube.

One of the tools typically used to describe chemical reactivity of molecules is the Fukui function.^119^ These are 3D functions of the position that show where the molecular electron density is changed most by adding an electron or a hole to the molecule. Such 3D information can also be summarized in a few numbers by performing an atomic population analysis of such functions, yielding the fractions of the added electrons and holes that localize on specific atoms in the system. We extended the latter analysis to the time domain, obtaining in this way the fraction of charge localized on atom X, with X = C, O, or H, and their sum for the entire CHO fragment, at a given instant. The time evolution of the Fukui-like charges is reported in Figure 4 for C, O, and H atoms in the case of the coupled Rh_19_–CHO and classical nanocube dynamics. What is more notable is that throughout the dynamics, oxygen species are made more electrophilic by the hole injection. Hydrogen, in contrast, holds a negligible fraction of the photoinduced charge, with minimal expected changes in its reactivity. In these conditions, oxygen has the tendency to acquire electrons, while hydrogen tends to release the electronic charge, becoming more attracted to the C atom, which is the closest electrophile. On the other hand, the oxygen atom is not attracted by the carbon atom, which is electron poor as well.

**Figure 5:**
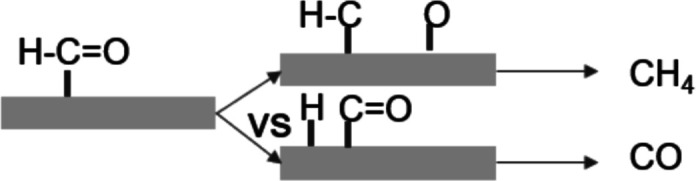
Scheme illustrating the fundamental steps that drive the reaction toward the production of CH_4_ or CO.

If we now consider the two competitive pathways leading from the CHO intermediate to CH_4_ and CO, respectively (see the scheme in Figure 5), we conclude that the path leading to CO, based on the reactivity of the H atom toward the Rh surface, is practically unaffected by the photoinduced electronic dynamics.

On the contrary, oxygen is made more reactive by the hole injection consequent to the light pulse, explaining how the latter enhances the probability of following the second (i.e., toward CH_4_) path. This is indeed the result experimentally observed.

Hole injection instead of electron injection on the CHO moiety may be considered a counterintuitive mechanism to favor a reduction reaction on the carbon atom. An analogous reaction, the Clemmensen reduction,^120,121^ leads to carbonyl hydrogenation catalyzed by amalgamated zinc in the presence of concentrated hydrochloric acid. In this case, two possible mechanisms have been proposed: the reaction goes on through direct protonation of the oxygen atom generating the carbanionic compound or through oxygen adsorption on zinc surface via carbenoid mechanism. In both cases, the electrophilicity of the oxygen atom increases, favored by the acidity of the solution, and the reaction is driven toward carbon–oxygen bond weakening. In the hydrogenation reaction of carbon dioxide catalyzed by rhodium nanocubes, the electrophilicity of the oxygen atom is favored by hole injection, which makes it more reactive toward other species and inclined to break the bond with the carbon atom that brings a hole excess too.

Summarizing, shining light on the CHO fragment adsorbed on the Rh nanocubes generates an overall hole population on C and O atoms, which leads to the selection of methane formation against carbon monoxide. The highest value of Fukui-like fraction for oxygen along the dynamics represents a further confirmation that the O atom looks for electron excess along the Rh surface.

So far, these findings concern a coherent electron dynamics that takes place within ≈50 fs from the pulse maximum. Electronic dephasing times due to the surrounding environment are on a similar time scale.^122^ Therefore, on the one hand, the reported results are not expected to change drastically when environment-induced electronic dephasing is accounted for. On the other hand, it is relevant to assess what the possible effects of such decoherence may be. To this end, we have performed test simulations by including environment-induced electronic decoherence by employing an approach based on the stochastic Schrödinger equation in its Markovian formulation; see the Supporting Information for details. To establish a limit behavior of dephasing, a fast dephasing time is applied, i.e., 5 fs. In these conditions, photoinduced charge injection becomes a transient process, with the charge going to zero at the end of the pulse. However, as shown in Figures S4 and S5 of the Supporting Information, the behavior of photoinduced charge populations is the same as in the simulations performed without decoherence. As such, while one can expect that decoherence may quantitatively affect selectivity, it does not qualitatively change the underlying explanation of the reaction selectivity.

The role of nuclear dynamics in the present photoinduced conditions is taken into account by analyzing the geometry of a simplified system, the positively charged acetaldehyde. A hole was created in the HOMO – 1 orbital (see the Supporting Information for details) to reproduce the excess positive charge on the carbon and oxygen atoms. We observe that the CO bond distance increases by 0.05 Å (4%) when the hole is created in HOMO – 1. This analysis provides evidence that, if activated by the interaction with light and with the time-dependent polarization of the NP, a weakening of the CO bond occurs, supporting the conclusions formulated by looking exclusively at the dynamics of the hot carriers.

## Conclusions

In this work, we identified the reason for selectivity enhancement toward CH_4_ against CO in the CO_2_ reduction, photo-assisted by the Rh nanocube, by studying the light-induced hot-carrier dynamics at the rate-determining step of the reaction. Charge injection dynamics in this multiscale treated CHO-rhodium nanocube revealed hole injection from Rh atoms to CHO fragment, with both carbon and oxygen atoms becoming electron deficient. This effect is enhanced when the full system is considered. Since both oxygen and carbon are electron deficient, while the hydrogen in the fragment acquires a small partial electron charge, the C–O bond is weakened, while the C–H is maintained. This selects the reaction path which leads, (almost) exclusively, to CH_4_ production against the formation of CO, made unlikely by the contemporary hole excess on C and O.

Moreover, even though the calculations performed in the absence of the classical portion of rhodium NP revealed two possible hole injection mechanisms, a direct one promoted by the light and an indirect one where holes generated on Rh atoms move toward CHO, when the full system is taken into account the direct mechanism becomes prevailing. The present work shows the potential of multiscale modeling to clarify the complex mechanisms of photocatalytic reactions based on metallic nanostructures, opening the way to the systematic rational design of these reactions.

## Methods

In order to provide an accurate and physically meaningful description of the electron dynamics occurring in the composite system with an external pulse, a time-resolved approach is mandatory. Our time-domain QM/continuum model describes the time evolution of the molecular density, via the appropriate formulation of the TDSE, coupled to a classical electromagnetic solver, based on the boundary element method (BEM),^123^ for the NP polarization. The continuum part is modeled on the time-resolved extension of the PCM, i.e., TD-PCM.^123^ Dielectric function of the NP is fitted from experimental data.^94^ Details about the QM/continuum partition are given in the Supporting Information.

A computationally convenient time-resolved version of BEM has been recently proposed,^92^ where the set of apparent charges on the NP surface becomes time-dependent. This makes the time-resolved BEM a powerful tool for describing the time evolution of a QM system interacting with a NP, giving rise to a model that can be dubbed TD-PCM-NP.^95^ The method employed has been developed in the quasi-static limit.

Within the TD-PCM-NP, the time-dependent wave function of the QM portion, |Ψ_S_(*t*)⟩, is propagated using TDSE
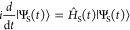
1with
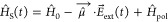
2where  is the field-free electronic Hamiltonian,  is the system dipole, and  is the polarization interaction term
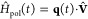
3where  is the generic electrostatic potential operator evaluated at the NP surface representative points. The electrostatic potential, which polarizes the NP, originates from the external field  and from the molecular density. The time-dependent system wave function |Ψ_S_(*t*)⟩ is expanded using the eigenstate basis {|λ⟩} of an effective field-free Hamiltonian, (92,93,96)
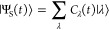
4with λ running over the electronic eigenstates, and *C*_λ_(*t*) are time-dependent coefficients evolving according to the Hamiltonian . Charges **q**_GS_ are obtained by a self-consistent calculation for the QM ground-state in the presence of the polarizable NP.

The set of |λ⟩ states is composed of the DFT ground state and GW-BSE derived excited states.^96^ The GW-BSE active space allows us to overcome the limitations of the implementations that employ time-dependent configuration interaction singles and time-dependent DFT in terms of achievable accuracy without compromising the accessible molecular sizes. Indeed, the GW-BSE approach is suited for an accurate description of electronic level alignments, charge transfer, and optical excitations in both extended and low dimensional systems with excitation energies and transition dipoles in line with high level theoretical chemistry methods.^124,125^

To obtain the |λ⟩ states, following ref (97), first the GW-BSE effective two-particles Hamiltonian for the Rh_19_–CHO cluster is diagonalized within the Tamm–Dancoff approximation. This provides a set of excitation energies, and
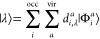
5with |Φ_*i*_^*a*^⟩ the Slater determinant for which one electron is promoted from an occupied MO *i* to a virtual one *a*, so that
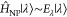
6with energy referred to the ground state Slater determinant |0⟩. The sums in eq 5 run over occupied (*i*) and virtual (*a*) MOs for a given spin state σ. Following the equilibration procedure, described in ref (97), that takes into account the mutual NP/Rh_19_–CHO polarization in the absence of an external field, the coupled NP-QM system dynamics is carried out.

The time-dependent PDOS_K_(*t*,ϵ) for the fragment *K* is defined as the expectation value with respect to |Ψ_S_(*t*)⟩ of the number operator  weighted by *w*_*i*_^K^ Lowdin weights. In our case, the fragments are either two (CHO and the Rh cluster) or six (C, O, H, rhodium atom closest to O, rhodium atom closest to C, and all the other Rh atoms). We are interested in the differential PDOS, ΔPDOS_K_(*t*,ϵ) = PDOS_K_(*t*,ϵ) – PDOS_*ini*_(ϵ), which is explicitly given by^126^
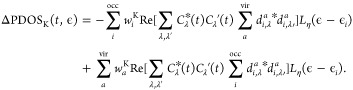
7*d*_*i*,λ_^*a*^ are the linear coefficients of the expansion for state λ (λ′) in eq 5, and *L*_η_ is a Lorentzian function *L* centered on MO energies ϵ_*i*_, with width η is used to obtain a smooth profile.

Assuming that the wave function at the initial time is the ground state of the system, i.e., |Ψ_S_(*t* = 0)⟩ = |0⟩ the initial PDOS_ini_(ϵ) is defined as
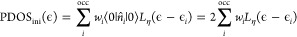
8

Two-dimensional maps of ΔPDOS_K_(*t*, ϵ) are reported in the Supporting Information.

Charge population is defined as
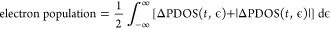
9
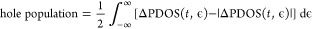
10ΔPDOS maps as a function of time (Figures S6 and S7) and computational details are given in the Supporting Information.

## Supporting Information Available

Multiscale modeling of the system, estimation of % contributions of the indirect and direct charge transfer, role of electronic dephasing, time evolution of ΔPDOS, and computational details (PDF)
