# Correction to “Peeking into the Femtosecond Hot-Carrier Dynamics Reveals Unexpected Mechanisms in Plasmonic Photocatalysis”

**DOI:** 10.1021/jacs.4c10033

**Published:** 2024-12-30

**Authors:** Giulia Dall’Osto, Margherita Marsili, Mirko Vanzan, Daniele Toffoli, Mauro Stener, Stefano Corni, Emanuele Coccia

Page 2214. In the next-to-last paragraph of the Results and Discussion, the sentence, “In these conditions, photoinduced charge injection becomes a transient process, with the charge going to zero at the end of the pulse.” should be replaced by the following: “In these conditions, erasing the electronic coherence highlights the slow nonradiative decay induced by the NP (Figure S4 of Supporting Information).”

The simulated plots showing the effect of dephasing in our original Figures S4 and S5 in the Supporting Information were erroneous due to a bug in the postprocessing code computing the time evolution of charge populations. We now provide corrected versions of Figures S4 and S5, which show plots obtained with the correct code.

Indeed, a pure electronic dephasing, i.e. an interaction channel between system and environment which only affects the relative phase between electronic states, is not expected to change the population of the states, and in turn the charge population. The original Figure S4 showed a transient charge populations (“photoinduced charge injection becomes a transient process, with the charge going to zero at the end of the pulse” in the original main text), which does not physically reflect the simulated process.

The following two paragraphs have been added to the updated Supporting Information (page 8):

“Figure S4 shows the charge population (in %) calculated on individual atoms (C, H, and O of the CHO fragment, and two rhodium atoms, panels a)-e)) as a function of time, in the presence of the external pulse and a dephasing time equal to 5 fs. Because of the fast dephasing source, electronic coherence is erased; however, the order of magnitude of charge population in the various cases is maintained, and one can see now the slow decay due to the plasmonic nanoparticle, which is instead “hidden” when electronic coherence (no applied dephasing) is still in the system. Indeed, a pure electronic dephasing, i.e. an interaction channel between system and environment which only affects the relative phase between electronic states, is not expected to change the population of the states, and in turn the charge population. The observed slow decay is not due to the *T*_2_ dephasing time to the time-dependent wavefunction of the QM subsystem, but to the decay channel activated by the nanoparticle.

The picture formulated in the main text concerning the coherent dynamics is also preserved in the case of dephasing, as shown in table f) in Figure S4 and in Figure S5, which shows the atomic charge fraction in the first 50 fs of the dynamics with dephasing: also in this case the oxygen atom is the most reactive, i.e. it makes it more likely that the reaction will move towards the formation of methane.”

The changes in the Supporting Information do not alter or impact the conclusions of the reported study.

## Supporting Information Available

Multiscale modeling of the system, estimation of % contributions of the indirect and direct charge transfer, role of electronic dephasing, time evolution of ΔPDOS, and computational details (corrected) (PDF)

