# Supplementary material for: Peeking into the Femtosecond Hot-Carrier Dynamics Reveals Unexpected Mechanisms in Plasmonic Photocatalysis
Source: J Am Chem Soc. 2024 Jan 10;146(3):2208–18. doi: 10.1021/jacs.3c12470 (PMC10811681; doi:10.1021/jacs.3c12470)
Supplement: Supplementary file 1 — ja3c12470_si_001.pdf [file ja3c12470_si_001.pdf]

Supplementary Information for "Peeking into the femtosecond hot-carrier dynamics reveals unexpected mechanisms in plasmonic photocatalysis"

Giulia Dall'Osto,[†] Margherita Marsili,[*,‡] Mirko Vanzan,[¶,†] Daniele Toffoli,[§] Mauro Stener,[§] Stefano Corni,[†,‖] and Emanuele Coccia[*,§]

†*Dipartimento di Scienze Chimiche, Università di Padova, via F. Marzolo 1, 35131, Padova, Italy*

‡*Dipartimento di Fisica e Astronomia "Augusto Righi", University of Bologna, Viale Berti Pichat 6/2, 40127, Bologna, Italy*

¶*Dipartimento di Fisica, University of Milan, Via Giovanni Celoria 16, 20133, Milano, Italy*

§*Dipartimento di Scienze Chimiche e Farmaceutiche, University of Trieste, via L. Giorgieri 1, 34127, Trieste, Italy*

‖*Istituto Nanoscienze-CNR, via Campi 213/A, 41125, Modena, Italy*

E-mail: margherita.marsili@unibo.it; ecoccia@units.it

Effect of cutting a classical NP

As mentioned in the main text, performing full QM electrodynamics calculations on large nanoparticles (NPs) is not feasible, and for this reason we split the rhodium nanocube into

an extended part described classically, and a small vertex treated at QM level. We have carefully studied the system to ensure the reliability of our approach. Below we report some results of our investigation: i) the comparison between the absorption spectra of a QM cluster and classical clusters modeled in different ways but with similar dimension, ii) the comparison of the absorption spectra of the NP with either a classical or an atomistic cluster as vertex, iii) the comparison of the potential generated by the NP with either a classical or an atomic cluster as vertex.

Taking on that calculations on a QM nanocube with dimensions in the order of tens of nanometres are unfeasible, we decided to compare the response of a smaller cluster when it is treated either at QM or classical level. The atomistic Rh_{19} cluster has been obtained by cutting a rhodium slab along the 111 direction, obtaining a two-layer cluster. This structure is the same employed in the main text as the nanocube vertex bonded to the CHO residue. Its absorption spectrum has been computed at BSE level of theory using MolGW code on the basis of 400 electronic states below 5 eV. On the other hand, differently shaped clusters treated classically have been built following various strategies. One structure is built connecting the position of atoms in the Rh_{19} cluster multiplied by a factor useful to have a volume equal to the cluster volume estimated on the basis of the rhodium Van der Waals radius (Figure S1a) or the Wigner-Seitz radius (Figure S1d). Other structures have been obtained as continuation of the cut nanocube reported in the main text considering either a rounded (Figure S1b) or a planar (Figure S1c) vertex. In the framework of boundary elements methods (BEM),[1] the surface has been tasselled by different number of tesserae (from 330 to 676) and metallic nature has been accounted by means of the Palik dielectric function of rhodium.[2] The absorption spectrum has been computed as the Fourier Transform of the dynamics of the surface charges on the cluster through the IEF-PCM equation in time domain[3,4] applying a Gaussian electric field oriented perpendicular to the cluster planar surface.

The absorption spectra (Figure S1e), reported in terms of the imaginary part of the

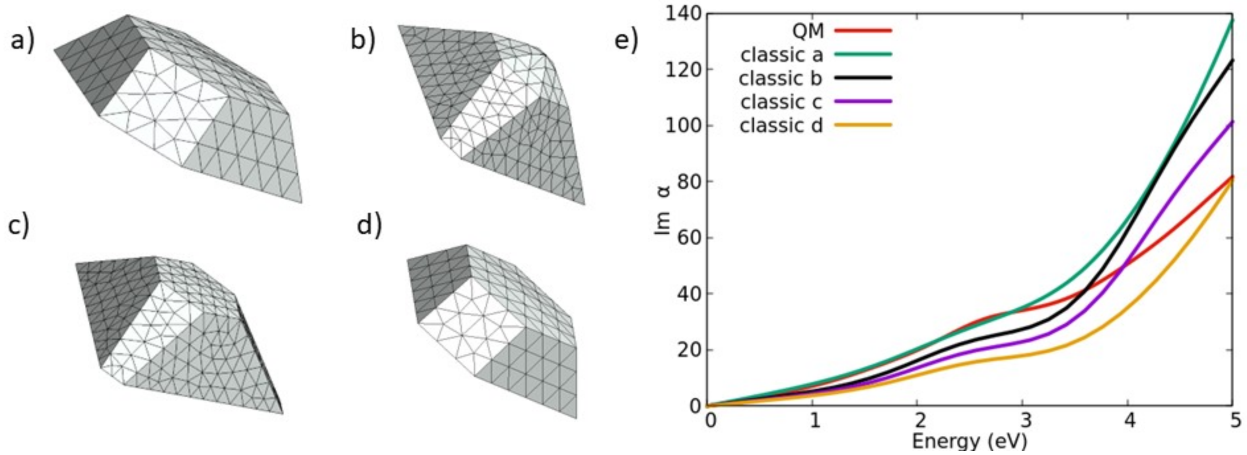

Figure S1: Structure of classical clusters built connecting the centre of the atoms position multiplied by the Van der Waals radius (a), built with a rounded vertex as continuation of the cut nanocube (b), built with a planar vertex as continuation of the cut nanocube(c), built connecting the centre of the atoms position multiplied by the Wigner-Seitz radius (d). e) Comparison between the absorption spectra of the quantum cluster (red line) and the absorption spectra computed at classical level for four different structures reported in panels (a-d).

polarizability, have the same trend both in terms of intensity and profile comparing the QM results and those of different atomistic clusters, meaning that the electrodynamics response is quite similar. In particular, in the frequency region interesting for the present work, around $2.5 - 3.5$ eV (where the pulse wavelength lies), there is a good agreement between the spectrum of the QM cluster and the classical one built on the basis of the Van der Waals radius (structure a). Moreover, the results point up that the absorption spectrum computed classically depends on the way the structure is built: structures a and d which have same shape but different dimension show similar spectra but with different intensity, as well as structures b and c have common features in the absorption spectra but slightly different intensities since structure b has a larger volume.

Moreover, we compared the absorption spectra of the cut nanocube close to the QM cluster, the cut nanocube alone and the full NP (in which the nanocube and the vertex are a single body treated classically). As Figure S2 shows, the absorption spectra of the three structures are perfectly superposed, since the structural differences are too small to be detected. This result points out that from the NP point of view, the dynamics in the three

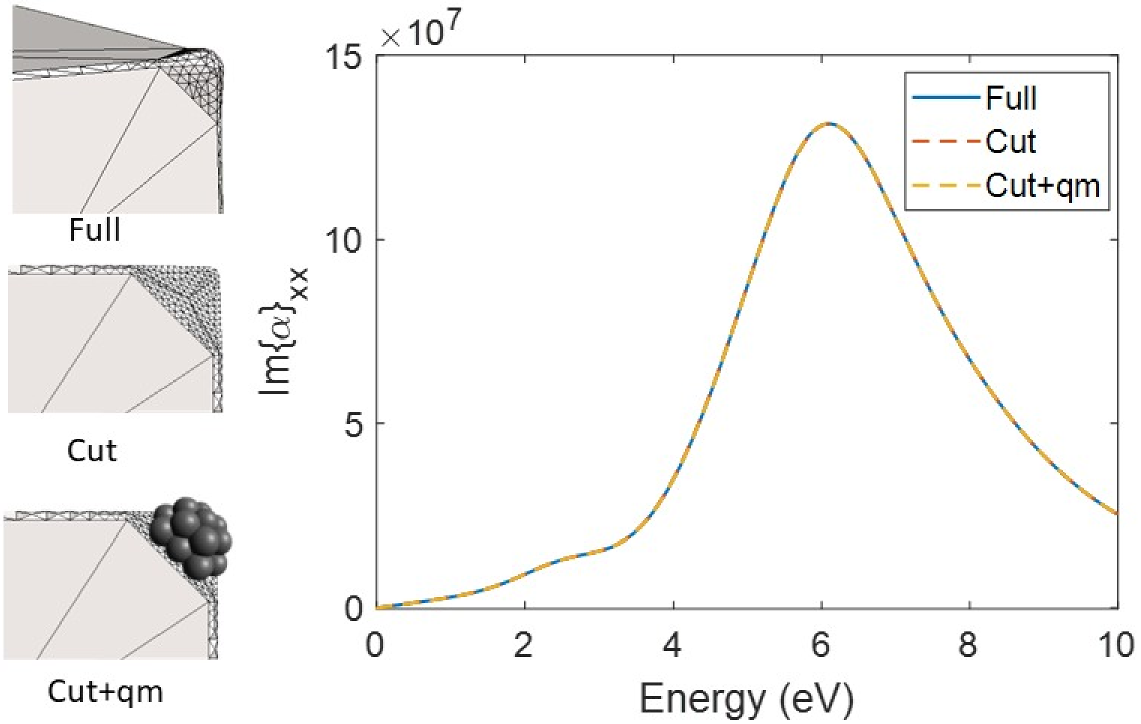

Figure S2: Comparison between the absorption spectra of the nanocube close to the quantum cluster, the cut nanocube alone and the full NP. The three structures are reported on the left side of the Figure.

different situation are quite close.

Finally, in order to investigate how the cut close to the nanocube vertex impacts on the NP response we computed the potential generated on a surface opposite to the atomistic vertex when close to the full NP (i.e. with the vertex "attached" to the cut surface) and in proximity to the cut nanocube close to a classic vertex (i.e. the vertex and the cut surface are spaced by the rhodium inter-layer distance). We report in table S1 the potential computed on the centre of the surface (at coordinates [0,0,0] Å) which is a point laying on the line perpendicular to the cut vertex and on one corner of the surface (at coordinates [6,3.5,6] Å). The results show a very good accordance in terms of potential, and they are very close in particular in the lateral point of the surface (at coordinates [6,3.5,6] Å) while small discrepancies can be noticed on the potential computed in the central point that is more affected by the difference on the vertex in the two structures due to the vicinity. The overall effect of the two opposite surfaces generated when interfacing the cut NP with a

classical vertex is not strong, at least in terms of generated potential and thus this structure can be safely employed for further calculations.

Table S1: Potential in atomic units generated on two points close to the NP vertex computed at two frequency (2.7 eV and 3.4 eV) assuming the full NP (i.e. the vertex is attached to the cut NP) and the cut NP put close to the cut vertex.

Structure	V([0,0,0] Å) 2.7 eV	V([6,3.5,6] Å) 2.7 eV	V([0,0,0] Å) 3.4 eV	V([6,3.5,6] Å) 3.4 eV
3* ▫`results_si/full.jpg`	-1818.38	-1504.82	-1975.20	-1625.55
3* ▫`results_si/cut+vdw.jpg`	-1715.43	-1555.20	-1769.81	-1664.25

Indirect vs direct charge-transfer mechanism

To discriminate the percentage of direct and indirect charge-transfer mechanism in CHO we fitted the hole population in CHO with two sigmoid functions respectively in the regions where the two mechanisms are identified: fitting in 0-42 fs to get the direct mechanism percentage, and in 43-66 fs to get the indirect mechanism percentage. Moreover, also the hole population in Rh fragment has been fitted with a sigmoid function in the range 43-66 fs when indirect charge transfer prevails. The fitting functions and the relative equations are reported in Figure S3. The equation in panel b shows that the sum of the direct and indirect mechanism percentage is 4.0159 % (the sum of the gap highlighted by the second sigmoid, 1.2215, and the minimum of the sigmoid, 2.7944). Dividing the gap individuated by the sigmoid in panel a (3.0257 %) by the maximum hole population (4.0159 %), we can estimate that the direct mechanism is responsible for the 75 % of the hole injected in CHO (as the hole population increase during the presence of the field) and the remaining 25% of the hole population is created through indirect charge transfer from Rh atoms after the end of the incident field. Moreover, also the hole population variation in Rh fragment in the range 43-66 fs (1.18 %) mirrors the increase of hole population in CHO fragment (1.22 %).

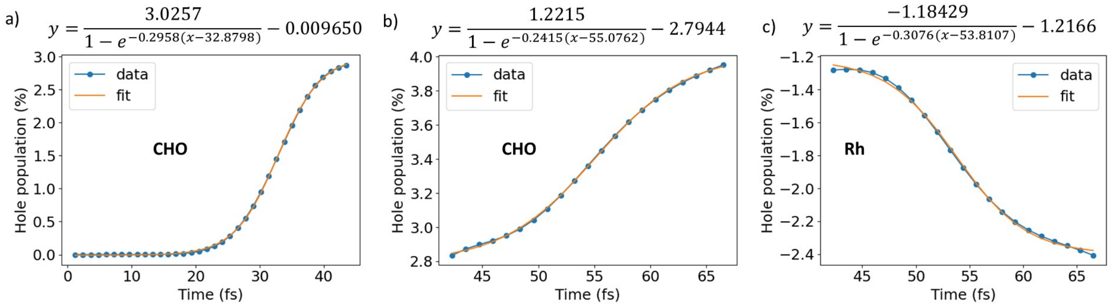

Figure S3: Fitting of CHO hole population reported in Figure 2b of main text, in the range 0-42 fs (panel a) and 43-66 fs (panel b). Fitting of hole population in Rh fragment in the range 43-66 fs (panel c). The equation of the fitted curves are reported above the corresponding plot.

Role of electronic dephasing

Electronic dephasing is included in TD-PCM-NP by means of the theory of open quantum systems. In the jargon of the open quantum systems theory, the degrees of freedom that we treat explicitly are the electronic ones of the QM portion. Whatever is not included in this definition is considered as environment. Such implementation[5,6] is based on the stochastic formulation of the time-dependent Schrödinger equation (SSE).[7] SSE in the Markovian limit is given by:[7]

$$i\frac{d}{dt}|\Psi_S(t)\rangle = \hat{H}_S(t)|\Psi_S(t)\rangle + \sum_q^M l_q(t)\hat{S}_q|\Psi_S(t)\rangle - \frac{i}{2}\sum_q^M \hat{S}_q^\dagger \hat{S}_q|\Psi_S(t)\rangle, \tag{1}$$

where $\hat{S}_q$ describes the effect of the environment on the quantum system, through the M interaction channels q. The non-Hermitian term $-\frac{i}{2}\sum_q^M \hat{S}_q^\dagger \hat{S}_q$ describes the dissipation, whereas $\sum_q^M l_q(t)\hat{S}_q$ is the fluctuation, modeled by a Wiener process $l_q(t)$, both generated by the environment. The system coincides with the electronic degrees of freedom of the QM portion, affected by the classical NP. Populations and coherences of the states of the system at time t are obtained by averaging on the number of independent SSE realizations.[5]

The pure dephasing operator is defined as[5]

$$\hat{S}_q^{\mathrm{dep}} = \sqrt{\gamma_q/2}\sum_p P(\lambda', \lambda)|\lambda'\rangle\langle\lambda|, \tag{2}$$

where $P(\lambda', \lambda)$ is equal to -1 if $\lambda' = \lambda$ or equal to 1 otherwise. Dephasing time T_2 is equal to the inverse of γ_q, which is given as parameter. Since γ_q is the same for any state pair, $T_2 = \frac{1}{\gamma}$.

ΔPDOS applied to the SSE is

$$
\begin{aligned}
\Delta\text{PDOS}_K^{\text{SSE}}(t,\epsilon) &= -\sum_i^{occ} w_i^K \text{Re}\left[\sum_{\lambda,\lambda'} \overline{C_\lambda^*(t)C_{\lambda'}(t)} \sum_a^{vir} {d_{i,\lambda}^a}^* d_{i,\lambda'}^a\right] L_\eta(\epsilon-\epsilon_i) \qquad (3)\\
&+ \sum_a^{vir} w_a^K \text{Re}\left[\sum_{\lambda,\lambda'} \overline{C_\lambda^*(t)C_{\lambda'}(t)} \sum_i^{occ} {d_{i,\lambda}^a}^* d_{i,\lambda'}^a\right] L_\eta(\epsilon-\epsilon_i).
\end{aligned}
$$

SSE version of charge population is defined by using $\Delta\text{PDOS}_K^{\text{SSE}}(t,\epsilon)$.

Data shown in Figure S4 report that coherence plays a role in the charge transfer. If dephasing is included, $\Delta\text{PDOS}_K^{\text{SSE}}(t,\epsilon)$ is characterized by the trajectory average of the $C_\lambda^*(t)C_{\lambda'}(t)$ products (Equation 3), with $C_{\lambda'}(t)$ being the time-dependent coefficient of the expansion of the propagated wave function into the field-free eingenbasis. Applying dephasing by using T_2 erases coherence among any state pair,[5] resulting in a statistical collection of electronic states. Such a statistical collection produces an almost zero contribution to charge injection: charge injection is therefore a superstate effect, depending on the evolution of time-dependent wave function as a whole.

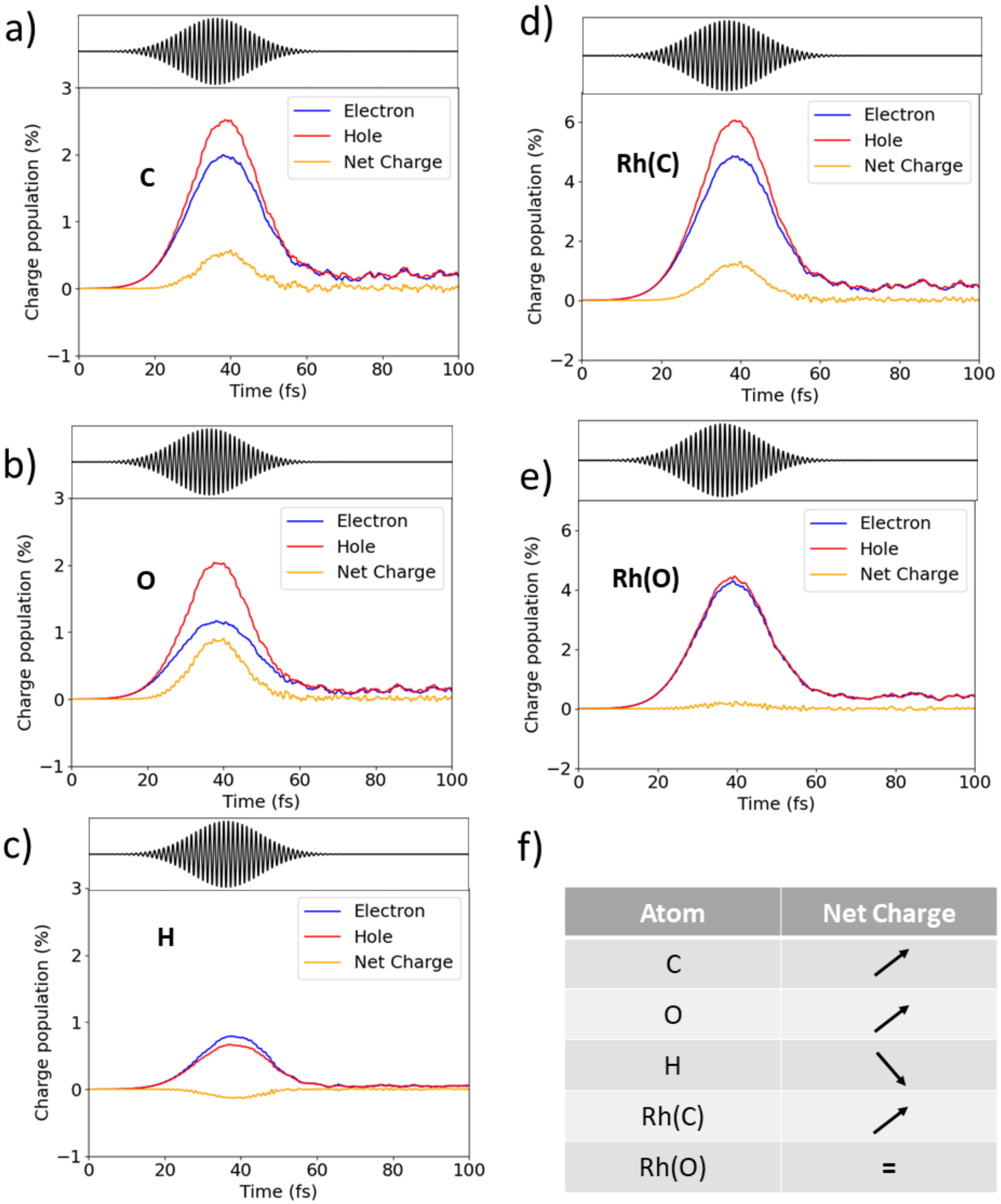

Figure S4: Electron, hole and net-charge injection for a) C, b) O, c) H, d) Rh closest to C, and e) Rh closest to O, from a SSE dynamics with $T_2 = 5$ fs. Panel f) summarizes the change of electron charge. The net charge is defined as the difference between hole and electron percentages.

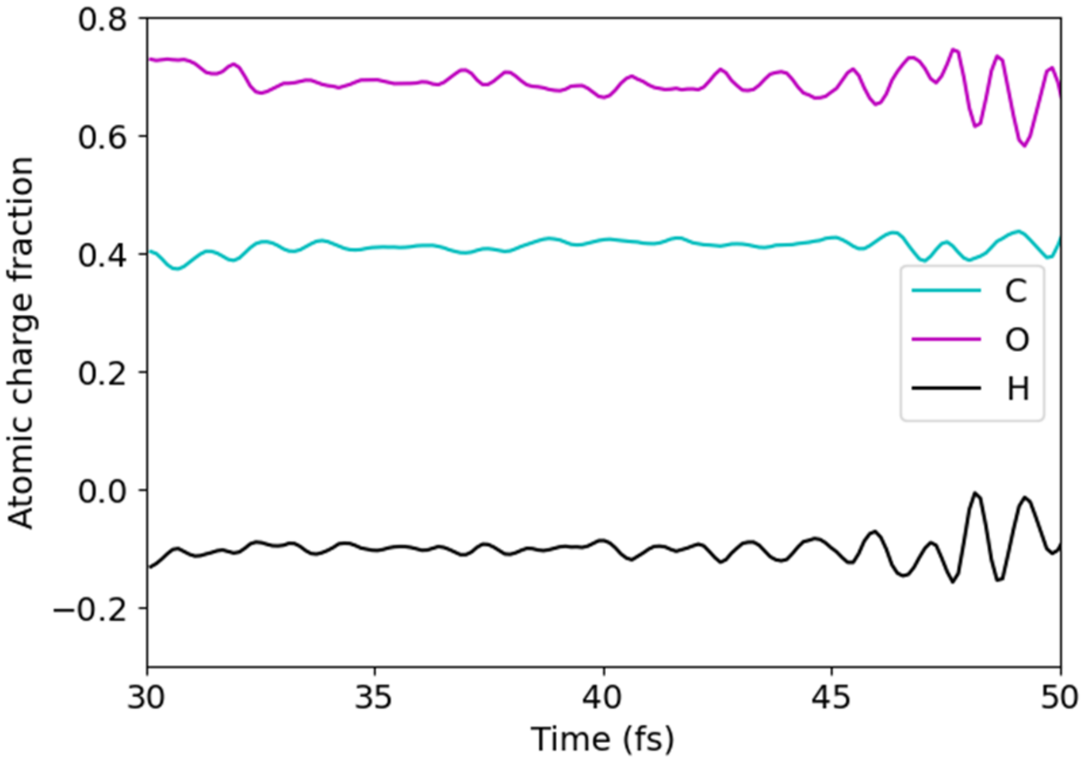

Figure S5: Time-resolved Fukui function for C, O and H atoms from the electron dynamics with $T_2 = 5$ fs.

Analysis of ΔPDOS maps

In the same conditions as those reported in the main text also the variation of the density of states has been computed, whose integration along the energy is reported in Figure 2 and Figure 3 of the main text. The time-dependent ΔPDOS has been computed as explained in Equation 7 of the main text, and it corresponds to the variation of the density of states as a function of the energy and time, with respect to the initial density of states. In the first case considered, the ΔPDOS has been computed for the QM portion alone (Rh_{19}-CHO) in presence of an incident electric field centered at 3.4 eV, which features, and time evolution are reported in Section 2 of the main text. We report two maps: one in the occupied band (between -3 and 0 eV) and one in the virtual band (between 3 and 7 eV). The results are reported in Figure S6. Panel a) and panel c) show that eight orbitals in the occupied band

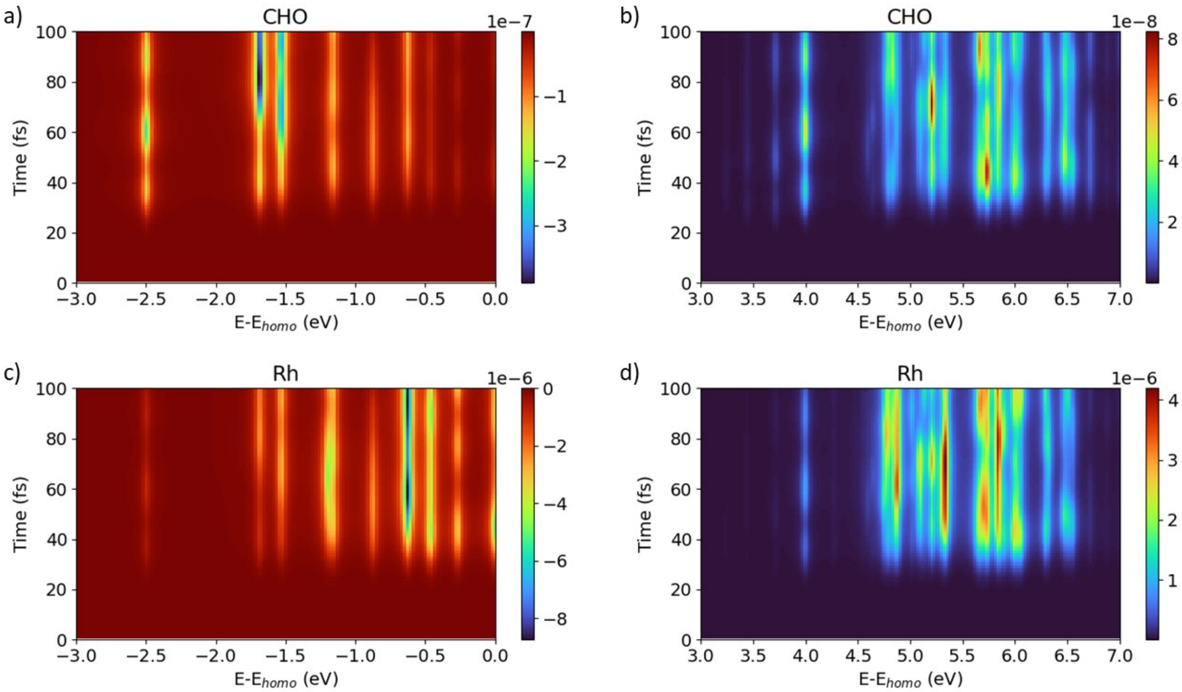

Figure S6: ΔPDOS of CHO (occupied band in panel a), virtual band in panel b)) and Rh (occupied band in panel c), virtual band in panel d)) fragments in absence of the NP.

range are involved in the dynamics after the pulse is switched on for both CHO and Rh fragments. Two orbitals, whose energies are -1.68 eV and -1.52 eV, are strongly emptied

in the CHO residue right after the end of the incident field evolution when the indirect charge transfer process is supposed to take place. These energies correspond to anti-bonding orbitals between carbon and oxygen atoms. Moreover, the orbital with energy at -0.62 eV that is quite completely localized on Rh atoms, is the most emptied in the occupied band of Rh residue during the interaction with the incident electric field. On the other hand, two virtual orbitals localized on CHO residue are mainly populated during the dynamics: one orbital with energy 5.74 eV is populated during the interaction with the pulse and another one with energy at 5.21 eV is populated when the pulse has already switched off. Both of them show an electron density between the carbon atom and the closest rhodium atom, as well as between the C-H bond.

The time dependent density of states have been reported in Figure S7 when the calculation is performed in presence of the NP, highlighting the occupied and virtual states localized on CHO or Rh_{19} residue. Comparing the results with those in absence of the classical NP, an enhancement of the maximum change in the state population can be noticed, as it has been highlighted also in the main text by the carrier injection values. The orbitals majorly involved during the dynamics are the same as in absence of the classical portion of the NP with some changes on the relative density of states: the occupied orbitals with energies at -1.68 eV and -1.52 eV are even more emptied than other occupied orbitals localized on CHO residue. On the other hand, the two states more populated during the dynamics are those with energy at 5.21 and 5.74 eV, but differently than in the previous case both of them are populated during the interaction with the incident pulse, as proof that the direct charge transfer is preferred and enhanced in presence of the NP as stated in the main text.

Computational Details

- The atomistic structure of Rh_{19} cluster used to model the corner of the Rh nanocube has been carved from a Rh(111) slab, optimized at the Density Functional Theory (DFT)

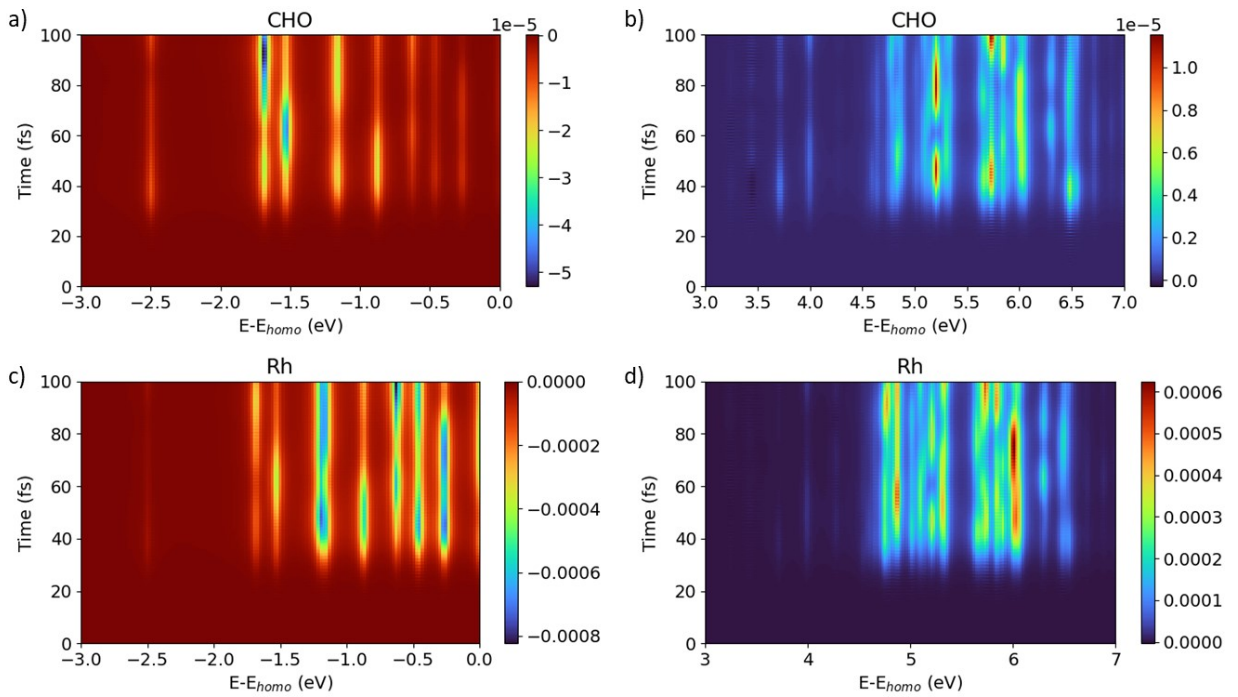

Figure S7: ΔPDOS of CHO (occupied band in panel a), virtual band in panel b)) and Rh (occupied band in panel c), virtual band in panel d)) fragments in presence of the classical NP.

using a plane-waves basis set as implemented in Quantum Espresso, version 6.3.[8,9] In this calculation electron-electron interactions were described by the Perdew-Burke-Ernzerhof (PBE) exchange-correlation functional,[10] core-electrons were modelled with the Optimized Norm-Conserving Vanderbilt (ONCV) pseudopotential[11] and the energy cut-off for the basis set expansion was set to 1080 eV. Previous calculations[12] performed on bulk Rh found an equilibrium lattice constant of 3.833 Å and such a parameter were used to build a 4-layers slab exposing a 4x4 Rh (111) supercell. The geometry optimization of this system was made on a 8x8x1 k-point grid and included an extra 20 Å vacuum layer added along the direction orthogonal to the surface and for dipole corrections,[13] both accounted to prevent the formation of spurious electric fields in the system. Keeping the two bottom layers fixed at their ideal bulk positions, all the remaining atoms were relaxed according to the Broyden–Fletcher–Goldfarb–Shanno (BFGS) quasi-Newton algorithm[14] until the residual forces were lower than 10^{-4} eV/Å,

with a convergence threshold on the ground state total energy of 10^{-9} eV. Once relaxed, Rh_{19} was extracted from the two outermost layers of the slab and then used as substrate for the geometry optimization of CHO species. Such a calculation was performed at the DFT level, using PBE XC-functional and a double-zeta basis set by means of AMS code,[15] freezing the bottom layer of the cluster in order to maintain Rh(111) surface structure.

- DFT and GW-BSE calculations were performed as implemented in the MOLGW code,[16] employing def2-TZVP basis set[17] and the PBE0 functional.[18] Eigenvalue-self-consistent G_nW_n calculations were carried out, with eigenenergies converging within 4 iterations. The contributions from core electrons were neglected in the calculation of the screened Coulomb interaction and of the single particle Green's function, whereas all the empty orbitals allowed by the basis set were considered for both. 455 $|\lambda\rangle$ states were employed for the Rh_{19}-CHO cluster dynamics.

- Electron dynamics, with and without the classical NP, have been carried out with WaveT code.[19,20] We have employed a Gaussian envelope function for the time-dependent external field:
$$\vec{\mathrm{F}}(t) = \vec{\mathrm{F}}_{\max} \exp\left(-\frac{(t-t_0)^2}{2\sigma^2}\right) \sin(\omega t), \tag{4}$$
where $\vec{\mathrm{F}}_{\max}$ is the field amplitude (the intensity I is equal to $\frac{1}{2}|\vec{\mathrm{F}}_{\max}|^2$), t_0 and σ are the center and the amplitude of the Gaussian respectively, and ω is the pulse frequency. Pulse wavelength: 365 nm (FWHM 21 fs), weak field (intensity of 3.3×10^4 W/cm^2), linear polarization perpendicular to the vertex of the nanocube. A second-order Euler algorithm[3] was used to propagate TDSE. We simulated 100 fs with a time step of 1.21 as.

- 764 SSE trajectories have been carried out, with a dephasing time T_2 of 5 fs, by using the dephasing operator defined in Ref. 5. All the other parameters are the same as used for the coherent dynamics. A combination of deterministic and random steps, via

the quantum jump algorithm,[5,21,22] has been employed to generate the uncorrelated SSE trajectories.

- A homemade Fortran 90 code has been used to compute the ΔPDOS as in Equation 7 of section Methods and its integral as in Equations 9 and 10 in order to compute electron and hole injection. The amplitude of the Lorentzian function is 0.0272 eV and the energy grid used to compute ΔPDOS and its integration goes from -21.7 eV to 21.7 eV with a step of 0.0217 eV. The post-processing calculation has been performed considering either two fragments (CHO and all rhodium atoms) or six fragments (C, O, H, Rh closest to O, Rh closest to C and all the other Rh atoms).

- The ionic configuration of acetaldeyde has been obtained by removing an electron from HOMO-1, which involves the C-O π bond. Geometry and SCF optimization and normal-mode analysis have been carried out at DFT/LDA level with VWN parametrization,[23] using a DZP basis set. The SCF has been run with unrestricted scheme. All the calculations have been carried out by means of the AMS code.[15] The same computational protocol has been used for the neutral acetaldeyde, except for the fact that a restricted SCF calculation has been carried out.

References

(1) Volakis, J. L.; Sertel, K. *Integral Equation Methods for Electromagnetics*; 2012; pp 1–392.

(2) Palik, E.; Gosh, G. *Handbook of Optical Constants of Solids*; Academic Press: San Diego, USA, 1998.

(3) Pipolo, S.; Corni, S. Real-Time Description of the Electronic Dynamics for a Molecule Close to a Plasmonic Nanoparticle. *J. Phys. Chem. C* **2016**, *120*, 28774–28781.

(4) Dall'Osto, G.; Gil, G.; Pipolo, S.; Corni, S. Real-time dynamics of plasmonic resonances in nanoparticles described by a boundary element method with generic dielectric function. *J. Chem. Phys.* **2020**, *153*, 184114.

(5) Coccia, E.; Troiani, F.; Corni, S. Probing quantum coherence in ultrafast molecular processes: An *ab initio* approach to open quantum systems. *J. Chem. Phys.* **2018**, *148*, 204112.

(6) Coccia, E.; Corni, S. Role of coherence in the plasmonic control of molecular absorption. *J. Chem. Phys.* **2019**, *151*, 044703.

(7) Biele, R.; D'Agosta, R. A stochastic approach to open quantum systems. *J. Phys. Condens. Matter* **2012**, *24*, 273201.

(8) Giannozzi, P. et al. QUANTUM ESPRESSO: a modular and open-source software project for quantum simulations of materials. *J. Phys. Condens. Matter* **2009**, *21*, 395502.

(9) Giannozzi, P. et al. Advanced capabilities for materials modelling with Quantum ESPRESSO. *J. Phys. Condens. Matter* **2017**, *29*, 465901.

(10) Perdew, J. P.; Burke, K.; Ernzerhof, M. Generalized gradient approximation made simple. *Phys. Rev. Lett.* **1996**, *77*, 3865.

(11) Hamann, D. R. Optimized norm-conserving Vanderbilt pseudopotentials. *Phys. Rev. B* **2013**, *88*, 085117.

(12) Vanzan, M.; Marsili, M.; Corni, S. Study of the Rate-Determining Step of Rh Catalyzed CO_2 Reduction: Insight on the Hydrogen Assisted Molecular Dissociation. *Catalysts* **2021**, *11*, 538.

(13) Bengtsson, L. Dipole correction for surface supercell calculations. *Phys. Rev. B* **1999**, *59*, 12301.

(14) D. Head, J.; Zerner, M. C. A Broyden—Fletcher—Goldfarb—Shanno optimization procedure for molecular geometries. *Chem. Phys. Lett.* **1985**, *122*, 264.

(15) Rüger, R.; Franchini, M.; Trnka, T.; Yakovlev, A.; van Lenthe, E.; Philipsen, P.; van Vuren, T.; Klumpers, B.; Soini, T. AMS 2022.1, SCM, Theoretical Chemistry, Vrije Universiteit, Amsterdam, The Netherlands. 2022; `http://www.scm.com`.

(16) Bruneval, F.; Rangel, T.; Hamed, S. M.; Shao, M.; Yang, C.; Neaton, J. B. MOLGW 1: Many-body perturbation theory software for atoms, molecules, and clusters. *Comput. Phys. Commun.* **2016**, *208*, 149.

(17) Pritchard, B. P.; Altarawy, D.; Didier, B.; Gibson, T. D.; Windus, T. L. New Basis Set Exchange: An Open, Up-to-Date Resource for the Molecular Sciences Community. *J. Chem. Inf. Model.* **2019**, *59*, 4814.

(18) Adamo, C.; Barone, V. Toward reliable density functional methods without adjustable parameters: The PBE0 model. *J. Chem. Phys.* **1999**, *110*, 6158.

(19) Coccia, E.; Fregoni, J.; Guido, C. A.; Marsili, M.; Pipolo, S.; Corni, S. Hybrid theoretical models for molecular nanoplasmonics. *J. Chem. Phys. 153*, 200901.

(20) Mennucci, B.; Corni, S. Multiscale modelling of photoinduced processes in composite systems. *Nat. Rev. Chem.* **2019**, *3*, 315.

(21) Dalibard, J.; Castin, Y.; Mølmer, K. Wave-function Approach to Dissipative Processes in Quantum Optics. *Phys. Rev. Lett.* **1992**, *68*, 580.

(22) Mølmer, K.; Castin, Y.; Dalibard, J. Wave-function Approach to Dissipative Processes in Quantum Optics. *J. Opt. Soc. Am. B* **1993**, *10*, 524.

(23) Vosko, S. H.; Wilk, L.; Nusair, M. Accurate spin-dependent electron liquid correlation energies for local spin density calculations: a critical analysis. *Can. J. Phys.* **1980**, *58*, 1200.
